# Supplementary material for: The genome sequence of the click beetle, Ampedus sanguinolentus sanguinolentus (Schrank, 1776)
Source: Wellcome Open Res. 2025 Feb 24;10:96. [Version 1] doi: 10.12688/wellcomeopenres.23712.1 (PMC11933788; doi:10.12688/wellcomeopenres.23712.1)
Supplement: Peer review history

# Reviewer report: Reviewer response for version 1

**DOI:** 10.21956/wellcomeopenres.26160.r119541

**Published:** 2025-06-13

**Authors:** Erich D Jarvis

**Affiliations:** 1 Rockefeller University, Millbrook, USA

Everything about the article looks great. The only issue I have is the Hi-C contact map of Figure 5. It does not look like a usual map, where you can see boundaries between chromosomes more cleaner than this. What are the two perpandicular white lines? Are you sure the settings are good? It would be useful to label the chromosomes.

Are sufficient details of methods and materials provided to allow replication by others?

Yes

Is the rationale for creating the dataset(s) clearly described?

Yes

Are the datasets clearly presented in a useable and accessible format?

Yes

Are the protocols appropriate and is the work technically sound?

Yes

Reviewer Expertise:

Genomics and Neuroscience

I confirm that I have read this submission and believe that I have an appropriate level of expertise to confirm that it is of an acceptable scientific standard.

# Reviewer report: Reviewer response for version 1

**DOI:** 10.21956/wellcomeopenres.26160.r120126

**Published:** 2025-03-24

**Authors:** Ruiqi Li

**Affiliations:** 1 University of Colorado Boulder, Boulder, Colorado, USA

Sivell presents a high-quality genome of the click beetle, *Ampedus sanguinolentus sanguinolentus. *I only have a few minor comments.

 1. The introduction did a great job describing click beetle's ecology, distribution, biology, etc. However, it would be great if the authors could provide another paragraph discussing what biological question we can use this genome to answer. See Li *et al *2024

 2. Figure1. It would be better if the authors could add a scale on the photo.

Are sufficient details of methods and materials provided to allow replication by others?

Yes

Is the rationale for creating the dataset(s) clearly described?

Yes

Are the datasets clearly presented in a useable and accessible format?

Yes

Are the protocols appropriate and is the work technically sound?

Yes

Reviewer Expertise:

Genomics

I confirm that I have read this submission and believe that I have an appropriate level of expertise to confirm that it is of an acceptable scientific standard.

## References

[1] : The genome sequence of the smooth giant clam, Tridacna derasa Röding, 1798. *Wellcome Open Res* .2024;9: 10.12688/wellcomeopenres.22618.1 375 10.12688/wellcomeopenres.22618.1 39429632 PMC11490831

[2] : The genome sequence of a heart cockle, Fragum fragum (Linnaeus, 1758). *Wellcome Open Res* .2024;9: 10.12688/wellcomeopenres.21134.1 129 10.12688/wellcomeopenres.21134.1 38989474 PMC11234083

# Reviewer report: Reviewer response for version 1

**DOI:** 10.21956/wellcomeopenres.26160.r120118

**Published:** 2025-03-06

**Authors:** Hume Douglas

**Affiliations:** 1 Agriculture and Agri-Food Canada, Ottawa, Canada

-Tribe should be spelled Ampedini

 -Not morphologically homogeneous especially after recognition of various groups of soft-bodied elateridae

 -I do not know of any evidence that adults mostly eat plants.

 -Still 17 extant subfamilies according to Bouchard et al.2024 (type genera), but not the same subfamilies now. This is a more current classification

 -What is an aberrant specimen?

 -Please mention that this genome could help with understanding elaterid phylogenetics and pest biology

 -iNaturalist suggests a strong peak of adult activity in May ( https://www.inaturalist.org/taxa/491379-Ampedus-sanguinolentus)

 -It would help to have a bit more explanation of Fig. 5. 

 This will be a useful contribution to beetle biology.

 Thanks,

 Hume Douglas

Are sufficient details of methods and materials provided to allow replication by others?

Yes

Is the rationale for creating the dataset(s) clearly described?

Yes

Are the datasets clearly presented in a useable and accessible format?

Yes

Are the protocols appropriate and is the work technically sound?

Yes

Reviewer Expertise:

Elateridae systematics

I confirm that I have read this submission and believe that I have an appropriate level of expertise to confirm that it is of an acceptable scientific standard.
